# Urban yellow fever outbreak—Democratic Republic of the Congo, 2016: Towards more rapid case detection

**DOI:** 10.1371/journal.pntd.0007029

**Published:** 2018-12-07

**Authors:** Brecht Ingelbeen, Nadine A. Weregemere, Harold Noel, Gaston P. Tshapenda, Mathias Mossoko, Justus Nsio, Axelle Ronsse, Steve Ahuka-Mundeke, Sandra Cohuet, Benoît I. Kebela

**Affiliations:** 1 Médecins sans Frontières, Kinshasa, Democratic Republic of the Congo; 2 European Programme for Intervention Epidemiology Training (EPIET), European Centre for Disease Prevention and Control (ECDC), Stockholm, Sweden; 3 Ministry of Health, Kinshasa, Democratic Republic of the Congo; 4 Direction des Maladies Infectieuses, Santé publique France, Saint-Maurice, France; 5 Direction de Lutte contre la Maladie, Ministry of Health, Kinshasa, Democratic Republic of the Congo; 6 Laboratoire de virologie, Institut National de Recherche Biologique, Kinshasa, Democratic Republic of the Congo; 7 Epicentre, Paris, France; University of Texas Medical Branch, UNITED STATES

## Abstract

**Background:**

Between December 2015 and July 2016, a yellow fever (YF) outbreak affected urban areas of Angola and the Democratic Republic of the Congo (DRC). We described the outbreak in DRC and assessed the accuracy of the YF case definition, to facilitate early diagnosis of cases in future urban outbreaks.

**Methodology/Principal findings:**

In DRC, suspected YF infection was defined as jaundice within 2 weeks after acute fever onset and was confirmed by either IgM serology or PCR for YF viral RNA. We used case investigation and hospital admission forms. Comparing clinical signs between confirmed and discarded suspected YF cases, we calculated the predictive values of each sign for confirmed YF and the diagnostic accuracy of several suspected YF case definitions. Fifty seven of 78 (73%) confirmed cases had travelled from Angola: 88% (50/57) men; median age 31 years (IQR 25–37). 15 (19%) confirmed cases were infected locally in urban settings in DRC. Median time from symptom onset to healthcare consultation was 7 days (IQR 6–9), to appearance of jaundice 8 days (IQR 7–11), to sample collection 9 days (IQR 7–14), and to hospitalization 17 days (IQR 11–26). A case definition including fever or jaundice, combined with myalgia or a negative malaria test, yielded an improved sensitivity (100%) and specificity (57%).

**Conclusions/Significance:**

As jaundice appeared late, the majority of cases were diagnosed too late for supportive care and prompt vector control. In areas with known local YF transmission, a suspected case definition without jaundice as essential criterion could facilitate earlier YF diagnosis, care and control.

## Author summary

Yellow fever is a mosquito-borne viral infection characterized by fever, followed after several days by jaundice, liver or kidney failure, shock or bleeding in up to 25% of cases. Although the virus primarily circulates in forests among primates, it can also be transmitted from human to human by mosquitoes in urban areas. If infected patients are detected early, they could benefit from timely supportive treatment, and control measures such as mosquito bite prevention, mosquito control, and mass vaccination campaigns, could prevent further spread of the disease. During 2015–16 a yellow fever outbreak spread in urban areas of Angola and DRC. The present study showed that most yellow fever patients that were diagnosed in DRC had travelled from Angola where they have been infected, and that most were adult men. Nevertheless, several patients have been infected locally, in urban settings in three provinces of DRC. Patients were diagnosed only when jaundice appeared, more than a week after their illness started, too late to fully benefit from supportive treatment. During urban outbreaks, improving early access to healthcare and earlier detection of patients by recognizing acute fever when malaria infection is excluded, could improve yellow fever care and control.

## Introduction

Yellow fever (YF) is a mosquito-borne viral infection characterized by an initial non-specific flu-like phase that lasts for 3 to 6 days and includes fever, headaches and myalgia. In 15%–25% of cases, a toxic phase follows with mild or severe jaundice, liver and kidney failure, which might lead to shock or bleeding [1,2]. No specific treatment exists. Approximately half of the severe cases lead to death within 7 to 10 days [2,3]. YF virus circulates primarily among forest-bound primates in a sylvatic cycle. Like other flaviviruses, YF can spread widely in urban areas, when transmitted from human to human by mosquito vector *Aedes aegypti* or potentially *Aedes albopictus* [4]. Female mosquitos become infected from a blood meal of an infected human. The incubation period in humans is 3–6 days [5]. Outbreak control relies on mosquito bite prevention, vector control, and mass vaccination campaigns. Early case detection by using an adapted case definition could allow earlier implementation of control measures for outbreak containment.

In the Democratic Republic of the Congo (DRC) each year a large number of sporadic YF infections occur following sylvatic transmission, when YF virus is transmitted by mosquitoes from non-human primates to persons living or working in forest areas [6]. Because of limited mobility of patients infected in forest areas, YF transmission rarely reaches urban environments.

In December 2015 YF cases were detected in the Angolan capital Luanda. In March 2016, the outbreak in Angola intensified, resulting in cases spreading to bordering provinces of DRC and its capital Kinshasa [7]. We carried out an investigation of the urban DRC outbreak to identify cases and describe the outbreak. Furthermore, we compared the performance of the case definition applied during the outbreak to alternative case definitions, aiming at an improved, timelier detection of cases in future urban outbreaks.

## Methods

### Study design and population

We present a detailed description of the 2016 YF outbreak in DRC and an analysis of the diagnostic accuracy of the case definition used, compared to alternatives. The YF cases related to this outbreak were reported to the national surveillance system between January and August 2016. In the analysis of the case definitions, we excluded vaccinated patients (at least ten days before symptom onset) and patients infected through sylvatic YF transmission (staying in a forest area in the two weeks to three days before symptom onset).

### Case definitions and YF confirmation

During the outbreak in DRC, the suspected case definition for routine surveillance in DRC was used, as also proposed in WHO guidelines [8]: an acute onset of fever followed by jaundice within 14 days after first symptoms onset. Any patient presenting at a healthcare facility meeting with this suspected case definition was notified to the Ministry of Health. Blood samples collected for every suspected case were tested for laboratory confirmation of yellow fever at the Institut National de Recherche Biomédicale (INRB), Kinshasa. A suspected case became confirmed when anti-YF IgM antibodies or YF viral RNA was detected in serum, if the patient was not immunized against YF. YF IgM detection consisted of an initial enzyme-linked immunosorbent assay (ELISA) to detect anti-flavivirus IgM antibodies, followed by a series of consecutive virus-specific ELISA tests to exclude other flavivirus infections such as Zika, dengue, and West Nile viruses. The ELISA results needed to be further confirmed by demonstrating a four-fold increase in YF neutralizing antibodies or by a Plaque Reduction Neutralization Test. Simultaneously a RT-PCR assay tested the presence of YF viral RNA in the blood sample. A suspected case was discarded when neither YF specific IgM antibodies nor YF viral RNA were detected. Confirmed cases were further classified as imported or autochthonous relying on travel history to Angola within two weeks to three days before symptom onset. Current or recent malaria (co-)infection was tested during July-August 2016 among patients admitted to a YF management facility, through detection of P. falciparum HRP-2 antigen (SD BIOLINE Malaria Ag P.f, Standard Diagnostics Inc.).

### Data sources

Patient demographics, symptoms, malaria co-infections, laboratory YF confirmation results, travel and vaccination history were extracted from case investigation forms (with suggested symptoms), patient medical files, and daily reports of notified suspected cases. Symptoms and malaria co-infections were systematically recorded in Kinshasa between 28 May and 02 August 2016, and thus only available for 14 confirmed cases and 97 discarded cases. GPS coordinates of places visited by patients while infectious, during the first 6 days after symptom onset, were used to map areas with possible ongoing transmission of YF.

### Data analysis

We described recorded characteristics and deaths as frequencies, percentages or medians with interquartile range. We compared differences in frequencies between cases by using Pearson’s Chi-squared test (or Fisher’s exact test, as appropriate) and differences in median age using the Wilcoxon rank-sum test (when not normally distributed). We used QGIS 2.18 with OpenStreetMap shapefiles to generate a geographical dot distribution map of cases in Kinshasa. To avoid revealing the exact locations of the cases, we rounded longitude and latitude coordinates to 10^−3^ degrees, to assign a random point location within a 110m radius of the patients’ recorded residences.

We identified potential predictors of YF by calculating positive and negative likelihood ratios (LR+ and LR-) for the presence or absence of every recorded symptom, severe anemia and a positive malaria test among confirmed and discarded cases for which those symptoms were recorded. LR+ is the increase in the probability of YF infection when the symptom is present, in other words sensitivity/(1-specificity) of that symptom to detect infection. LR- is the decrease in the probability of YF infection when the symptom is absent. We used combinations of predictive signs (LR+ or LR- larger than 2.5) to create new case definitions (four options). We drew receiver operating characteristic (ROC) curves to compare the diagnostic performance (sensitivity, specificity and Area Under the Curve (AUC)) of the DRC outbreak case definitions with our optional case definitions and those used in previous urban YF outbreaks in Uganda (2010/11), Brasil (2009) and Bolivia (1997/98) [9–11]. We performed analyses in R 3.4.1 and STATA 12.

### Ethics statement

The Ethical Review Committee at the University of Kinshasa approved the study (reference ESP/CE/049/2017). Only anonymized routine surveillance data, collected for the outbreak investigation was retrospectively analyzed. Therefore, no individual patient informed consent was asked.

## Results

### The yellow fever outbreak in DRC

**Fig 1:**
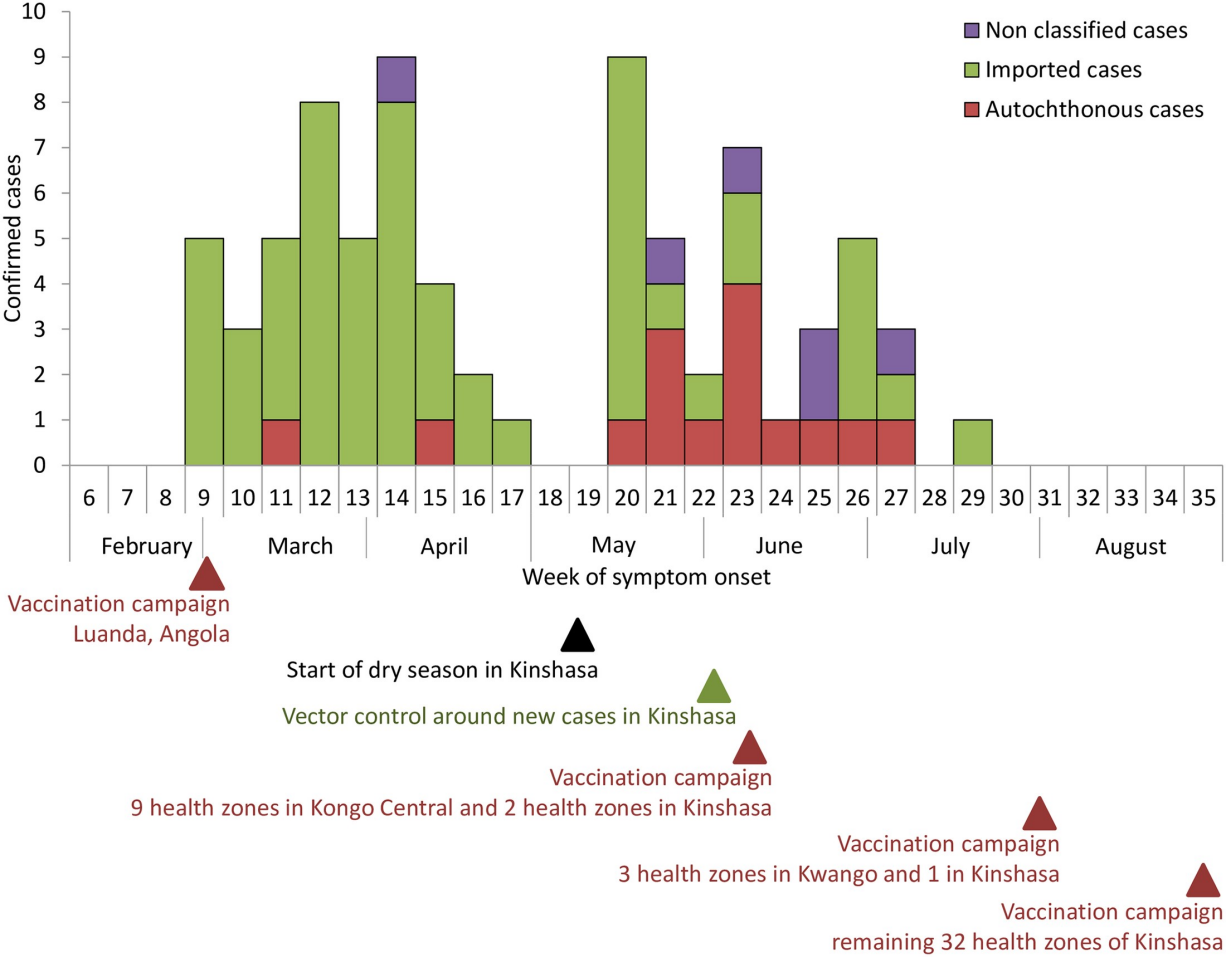
Weekly distribution of confirmed imported, autochtonous and non classified yellow fever cases, DRC, 2016.

Between 1 January and 11 August 2016, 2,269 suspected cases were reported in DRC. Of the 2,025 cases that underwent confirmatory testing, 78 (4%) were confirmed. Cases were confirmed in Kinshasa and two provinces neighbouring the Angolan border, Kongo-Central and Kwango. The first confirmed case had onset of symptoms on 22 February, the final case on 12 July (Fig 1). Of the 78 confirmed cases, 57 (73%) were imported from Angola. Imported cases occurred mostly among adult male patients (Table 1): 88% (50/57) men; median age 31 years (interquartile rate (IQR) 25–37). Fifteen (19%) YF confirmed case patients had not travelled to Angola, and acquired YF in urban settings in Kinshasa (n = 8), in the Angola-bordering Kwango (n = 4), and Kongo-Central (n = 3) provinces. Of these autochthonous cases 67% (10/15) were male; median age was 20 years (IQR 12–29; p<0.01). For six cases, no travel history could be retrieved (not classified). Six sylvatic cases, not related to this outbreak, were confirmed during the same period.

**Table 1:** Patient characteristics and outcome among imported and autochthonous confirmed yellow fever cases, DRC, 2016.

Characteristics	Imported (N = 57)		Autochthonous (N = 15)		p value
	n	%	n	%	
Aged ≤5 years	0	0.0	1	6.7	0.20
Male sex	50	87.7	10	66.7	0.05
Hospitalized	25 (N = 28)	89.3	7 (N = 8)	87.5	0.89
Died	17	29.9	1	7.1	0.08
Living in district where autochthonous cases have earlier been diagnosed	6	10.5	4	26.7	0.11

Mapping the imported and autochthonous confirmed cases led to the identification of one geographical cluster of three autochthonous confirmed cases occurring between 30 May and 10 June 2016 in the same neighborhood of Kinshasa, where case investigations revealed another 3 deaths of suspected cases with symptom onset in the same period (https://osf.io/tk3gn/). This cluster was linked to a previously unidentified case who had returned from Angola with a fever 22 days before (8 May). All other cases were widespread, and could not be linked to one another.

The median time from symptom onset to a first healthcare consultation in any healthcare facility was 7 days (IQR 6–9), to appearance of jaundice 8 days (IQR 7–11), to sample collection 9 days (IQR 7–14), and to hospitalization 17 days (IQR 11–26) (Table 2). The delay to sample collection was not significantly different (p = 0.88) among imported and autochthonous confirmed cases.

**Table 2:** Median delays between symptom onset and seeking healthcare, yellow fever diagnosis, hospitalization and death among confirmed yellow fever cases, DRC, 2016.

Delay from symptom onset to…	Median (days)	Interquartile range (days)
Healthcare consultation (N = 13)	7	6–9
Jaundice (N = 5)	8	7–11
Suspected case notification and sample collection (N = 77)	9	7–14
Hospitalisation (N = 4)	17	11–26
Death (N = 7)	15	10–16

Among the 74 confirmed cases tested by RT-PCR, 9 (12%) had detectable YF viral RNA. The blood samples of the 9 PCR-positive cases were collected at a median of 7 (range 1–14) days after onset of symptoms.

We recorded 18 deaths among confirmed cases, resulting in a case fatality of 23%. Confirmed cases died after a median of 15 days following the onset of symptoms.

### Symptoms

Symptoms and malaria (co-)infection were recorded for 14 confirmed and 97 discarded cases from Kinshasa. The median age of those confirmed cases was 24 years compared with 31 years among confirmed cases without recorded symptoms (p = 0.01); 64% and 86% (p = 0.06) were male, respectively. The 97 discarded cases had a median age of 15 years, compared with 16 years among discarded cases without recorded symptoms (p = 0.09); 53% and 56% (p = 0.36) were male, respectively.

Thirteen (92.9%) confirmed cases had fever and 10 (71.4%) had jaundice (Table 3). Of symptoms not included in the routine suspected case definition, myalgia, vomiting and headaches were most frequently reported, respectively among 88.9%, 77.8% and 66.7% of confirmed cases. One had hemorrhagic signs. We identified no confirmed cases with severe anemia at admission. Of 9 tested confirmed cases, 3 (33.3%) were malaria co-infected. None (0/3) showed rapid clinical improvement after starting antimalarial treatment.

**Table 3:** Percentage, frequency and predictive value of signs/symptoms reported among confirmed and discarded yellow fever cases, DRC, 2016.

Clinical sign	Confirmed cases		Discarded cases		p-value	Positive likelihood ratio (95%CI)	Negative likelihood ratio (95%CI)
	n/N	%	n/N	%			
**Fever**	13/14	92.9	93/97	95.9	0.61	1.0 (0.8–1.1)	1.7 (0.2–14.4)
**Jaundice**	10/14	71.4	93/97	95.9	<0.01	0.7 (0.5–1.0)	6.9 (2.0–24.6)
**Bleeding signs**	1/10	10.0	3/84	3.6	0.34	2.8 (0.3–24.4)	0.9 (0.8–1.2)
**Diuresis decrease**	4/10	40.0	7/80	8.8	<0.01	4.6 (1.6–12.9)	0.7 (0.4–1.1)
**Myalgia**	8/9	88.9	25/81	30.9	<0.01	2.9 (1.9–4.3)	0.2 (0.0–1.0)
**Headache**	6/9	66.7	46/81	56.8	0.57	1.2 (0.7–1.9)	0.8 (0.3–2.0)
**Nausea**	4/9	44.4	33/81	40.7	0.61	1.1 (0.5–2.2)	0.9 (0.5–2.0)
**Vomiting**	7/9	77.8	66/85	77.6	0.99	1.0 (0.7–1.5)	1.0 (0.3–3.6)
**Epigastric tenderness**	3/8	37.5	22/81	27.2	<0.01	1.4 (0.5–3.6)	0.9 (0.5–1.5)
Severe anaemia*	0/3	0.0	12/20	60.0	0.09	0.0	
Malaria HRP-2 positive**	3/9	33.3	73/90	81.1	<0.01	0.4 (0.2–1.0)	3.5 (1.9–6.6)
**Malaria HRP-2 positive with good response on treatment**	0/3	0.0	11/13	84.6	0.02	0.0	

* Severe anemia as defined by WHO: a hemoglobin level of below 80 g/l, or below 70 g/l for pregnant women and children between 6 and 59 months old.

** HRP-2 = histidine-rich protein II, an antigen expressed by *P*. *falciparum* trophozoites.

### Diagnostic performance of the suspected case definition

Also 88 (91%) discarded cases had both fever and jaundice, i.e. the DRC suspected case definition, resulting in a 9% specificity of the case definition. Considering that 1,947 of 2,025 tested suspects were discarded, it had a positive predictive value of 3.2%. Of 90 tested discarded cases, 73 (81.1%) were malaria positive. Malaria positive discarded cases had a median age of 12 (IQR 5–20) years, with 67% being under 18 years old. Malaria negative discarded cases were older (p = 0.03), median age 22 years (IQR 12–36), with 35% being under 18 years old. Of 13 malaria infected discarded cases, two (15.4%) did not improve after starting malaria treatment.

Decreased diuresis, myalgia and bleeding signs had the highest positive likelihood ratios, respectively 4.6, 2.9 and 2.8. Absence of malaria had the highest negative likelihood ratio, of 3.5.

**Fig 2:**
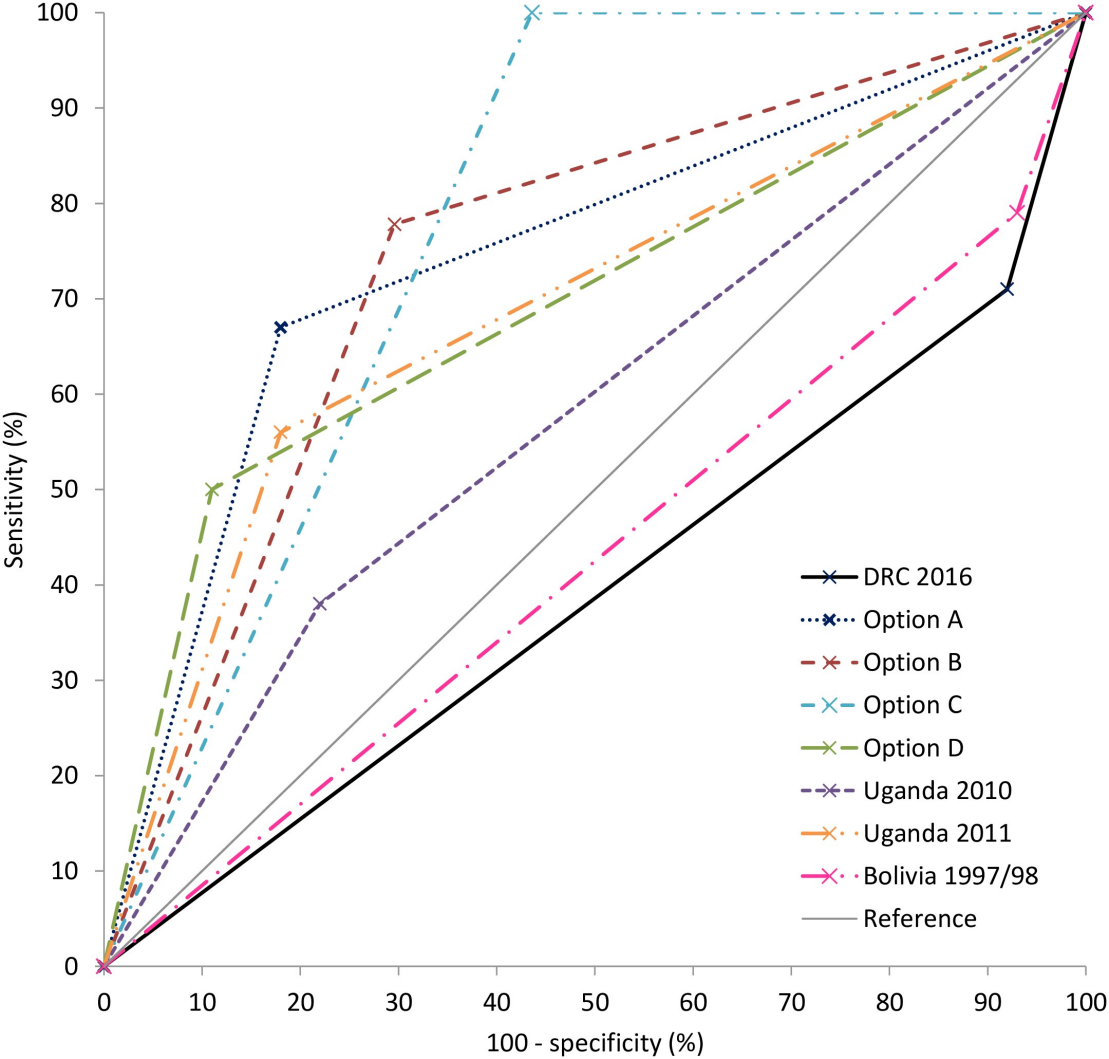
Receiver operating characteristic (ROC) curves of yellow fever suspected case definitions, applied on confirmed and discarded yellow fever cases with recorded clinical signs, DRC, 2016.

When comparing the DRC suspected case definition with four case definitions based on the most predictive and frequent signs (Options A, B, C and D), and case definitions used during urban outbreaks, we found that a combination of fever or jaundice and myalgia or a negative malaria test (Option C), yields the best combination of sensitivity (100%) and specificity (57%) resulting in an AUC of 0.78 (Fig 2 and Table 4). Other combinations with early symptoms result in lower sensitivity, but improved specificity. The two 2010/11 Ugandan outbreak case definitions had better specificity than the DRC case definition, but at the cost of lower sensitivity (AUC 0.58 in 2010 and 0.69 in 2011). The 1997/98 Bolivia case definition improved sensitivity (79%), but did not improve the specificity (7%; AUC 0.43). The 2009 Brazil case definition was not substantially different from that in DRC to allow comparison.

**Table 4:** Case definitions with sensitivity and specificity derived from the clinical signs of confirmed and discarded yellow fever cases, DRC, 2016.

Case definition	Description	Sensitivity (%)	Specificity (%)	Area under the curve (95%CI)
**DRC 2016**	Fever followed by jaundice (case definition applied during the DRC outbreak)	71	8	0.40 (0.27–0.52)
**Option A**	(Fever or jaundice) AND (malaria negative or not responding to malaria treatment)	67	82	0.74 (0.57–0.91)
**Option B**	(Fever or jaundice) AND myalgia	78	70	0.74 (0.59–0.89)
**Option C**	(Fever or jaundice) AND (malaria negative or myalgia)	100	57	0.78 (0.73–0.84)
**Option D**	(Fever or jaundice) AND (malaria negative or not responding to malaria treatment) AND (hemorrhage, decreased diuresis or myalgia)	50	89	0.69 (0.51–0.88)
**Uganda 2010** [10]	1^st^ stage (Nov-Dec) of the Uganda 2010/11 outbreak. Severe headache AND at least three of following: GI, dizziness, weakness, convulsions, unexplained bleeding	38	78	0.58 (0.39–0.77)
**Uganda 2011** [10]	2^nd^ stage (Jan-Feb) of the Uganda 2010/11 outbreak. Acute onset of fever AND (malaria negative or not responding to malaria treatment) AND (jaundice or unexplained bleeding)	56	82	0.69 (0.51–0.86)
**Bolivia 1997/98** [9]	Fever AND (jaundice or haemorrhagic symptoms or oliguria or death)	79	7	0.43 (0.32–0.54)

## Discussion

Although >2000 suspected YF cases were reported and tested in DRC, only 78 cases were confirmed, with symptom onset between February and July 2016. The peak of the YF outbreak in DRC followed and mirrored the ongoing outbreak in Angola, and started to wane as vaccination went on in Angola. The majority of confirmed YF affected young men from DRC working in Angola, who returned to DRC to seek healthcare following a YF infection contracted in Angola. Despite evidence of 15 locally transmitted cases in three different provinces, we observed no widespread urban YF transmission, as in Angola. Possible reasons for this might be (i) the timing of the first local transmission when the dry season took off, not allowing the vector carrying YF virus to replicate, (ii) the implementation of vector control measures around confirmed cases’ homes, or (iii) the mass YF vaccination campaigns before the end of the dry season in the affected health zones.

Patients were diagnosed too late for effective supportive care and to guide potential vector control measures. By the time infected patients with severe symptoms received appropriate healthcare (median time to hospitalization 17 days after symptom onset), the 12 to 15 critical days to prevent death through supportive care had already passed [2,3]. Several elements contributed to this delay: First, the majority of patients did not seek healthcare when going through the febrile phase of the disease within 5 days after symptom onset. YF testing is free in DRC but patients waited until symptoms worsened, afraid they might bear the cost of tests and treatments of other diagnosed conditions. Second, the suspected case definition applied during this outbreak encouraged notifying and testing only once jaundice appeared, 9 days after symptom onset. Finally, test results could take days to weeks because YF confirmatory testing was carried out in only one laboratory in the capital.

Low specificity of the DRC suspected case definition could only partially be explained by viral hepatitis. A 44% seroprevalence of viral hepatitis was found among suspected YF cases discarded during 2003–2012 in DRC [12]. Dengue virus RNA and chikungunya virus RNA were found in respectively 3.5% and in 0.4% of those discarded YF cases during the same period [13]. In our study, 81% of discarded cases were found to be malaria infected. Of those two thirds were children. This suggests that malaria may have been a leading cause of fever and jaundice among discarded cases in children.

A case definition in which jaundice would no longer be the main clinical criterion would allow more rapid detection of cases in districts where local transmission of YF is established. Nevertheless, considering that for each confirmed case another twenty suspected cases were notified and that no options for decentralized YF testing exist, other signs than fever are needed in the case definition to improve its specificity. Suspected case definitions used in previous urban YF outbreaks have relied on at least one severe sign occurring during the toxic phase of infection, and are therefore not more appropriate for timely diagnosis [9–11]. When comparing the performance of different case definitions applied on the confirmed and discarded cases in our study, “fever or jaundice, and myalgia or a negative malaria rapid diagnostic test (or blood slide)” (Option C) provided the most robust combination of sensitivity and specificity. Once index cases and clusters of local transmission are identified in an area using the DRC/WHO case definition, a switch to case definition option C in the area with established YF transmission could speed up the identification of YF cases. Ideally, the case definition we propose should be externally validated against clinical data from ongoing or future outbreaks in a similar urban context.

A limitation to the comparison of case definitions is that our reference group is composed of discarded cases, which met the suspected case definition. Those were likely not representative of the source population (any patient presenting at a healthcare facility), and therefore, the calculated specificities are probably underestimated, limiting the external validity of our estimates. Second, our study evaluated the diagnostic performance of the suspected case definition using symptoms of only 14 (out of 78) confirmed and 97 (of 1947) discarded cases with symptoms systematically recorded. Although age and sex distributions were slightly different of those of confirmed cases without recorded symptoms, we think this may be due to chance. We did not expect any differences in clinical presentation to occur among slightly older adult cases, or among cases reported earlier during the outbreak. Therefore, we assumed the frequencies of symptoms we reported, were representative of all cases. Our sample of cases was however too small for a precise estimate of the proportion of cases failing to respond to malaria treatment when malaria and YF co-infected. Finally, we were not able to quantitatively establish the improved timing of early diagnosis in our comparison of case definitions’ diagnostic performance, because only for jaundice the timing of onset was recorded. Recording the time of onset of each symptom could have allowed to compare the case definitions’ performance earlier through the course of the disease. Nevertheless, the case definition we proposed would probably have performed just as well when applied during the first days of illness, since fever and malaria infection would have been present already.

Due to the low accuracy of the case definition used during the 2016 YF outbreak in DRC and delays in accessing healthcare, most patients were diagnosed too late to receive beneficial supportive treatment and mitigate the complications of severe YF. Timely diagnosis of YF would also allow implementing vector control measures around confirmed cases’ homes to prevent further transmission. Improving early access to healthcare and developing case definitions that do not include jaundice as essential criterion, in areas where urban YF transmission is established, will facilitate early case detection and management.

## References

[1] Monath TP. Yellow fever: An update. Lancet Infect Dis. 2001;1: 11–20. 10.1016/S1473-3099(01)00016-0 11871403

[2] World Health Organisation. Yellow Fever Fact sheet (Updated May 2016) [Internet]. World Health Organization; 2016 [cited 8 Jul 2016]. http://www.who.int/mediacentre/factsheets/fs100/en/

[3] Johansson MA, Vasconcelos PFC, Staples JE. The whole iceberg: estimating the incidence of yellow fever virus infection from the number of severe cases. Trans R Soc Trop Med Hyg. NIH Public Access; 2014;108: 482–7. 10.1093/trstmh/tru092 24980556 PMC4632853

[4] Amraoui F, Vazeille M, Failloux AB. French Aedes albopictus are able to transmit yellow fever virus. Eurosurveillance. 2016;21: 14–16. 10.2807/1560-7917.ES.2016.21.39.30361 27719755 PMC5069433

[5] Monath T. Yellow fever: an update. Lancet Infect Dis. 2001;1: 11–20. 10.1016/S1473-3099(01)00016-0 11871403

[6] Garske T, Van Kerkhove MD, Yactayo S, Ronveaux O, Lewis RF, Staples JE, et al Yellow Fever in Africa: Estimating the Burden of Disease and Impact of Mass Vaccination from Outbreak and Serological Data. PLoS Med. 2014;11 10.1371/journal.pmed.1001638 24800812 PMC4011853

[7] World Health Organization. Yellow Fever Situation Report 7 October 2016 [Internet]. 2016. http://www.who.int/emergencies/yellow-fever/situation-reports/7-october-2016/en/

[8] World Health Organisation. WHO-recommended surveillance standard of yellow fever. In: WHO [Internet]. World Health Organization; 2015 [cited 20 Sep 2018]. http://www.who.int/immunization/monitoring_surveillance/burden/vpd/surveillance_type/passive/YF_standards/en/

[9] Van Der Stuyft P, Gianella A, Pirard M, Cespedes J, Lora J, Peredo C, et al Urbanisation of yellow fever in Santa Cruz, Bolivia. Lancet. 1999;353: 1558–1562. 10.1016/S0140-6736(99)03291-2 10334253

[10] Wamala JF, Malimbo M, Okot CL, Atai-Omoruto AD, Tenywa E, Miller JR, et al Epidemiological and laboratory characterization of a yellow fever outbreak in northern Uganda, October 2010-January 2011. Int J Infect Dis. International Society for Infectious Diseases; 2012;16: e536–e542. 10.1016/j.ijid.2012.03.004 22575876

[11] Mascheretti M, Tengan CH, Sato HK, Suzuki A, de Souza RP, Maeda M, et al Yellow fever: Reemerging in the state of Sao Paulo, Brazil, 2009. Rev Saude Publica. 2013;47: 881–889. 24626492 10.1590/s0034-8910.2013047004341

[12] Makiala-Mandanda S, Le Gal F, Ngwaka-Matsung N, Ahuka-Mundeke S, Onanga R, Bivigou-Mboumba B, et al High prevalence and diversity of hepatitis viruses in suspected cases of yellow fever in the Democratic Republic of Congo. J Clin Microbiol. 2017;33: JCM.01847-16. 10.1128/JCM.01847-16 28202798 PMC5405249

[13] Makiala-mandanda S, Ahuka-mundeke S, Abbate JL, Pukuta-simbu E, Nsio-mbeta J, Berthet N, et al Identification of Dengue and Chikungunya Cases Among Suspected Cases of Yellow Fever. Vector-borne Zoonotic Dis. 2018;XX: 1–7. 10.1089/vbz.2017.2176 29768102

